# Application of Machine Learning Algorithms for Tool Condition Monitoring in Milling Chipboard Process

**DOI:** 10.3390/s23135850

**Published:** 2023-06-23

**Authors:** Agata Przybyś-Małaczek, Izabella Antoniuk, Karol Szymanowski, Michał Kruk, Jarosław Kurek

**Affiliations:** 1 Department of Artificial Intelligence, Institute of Information Technology, Warsaw University of Life Sciences, 02-776 Warsaw, Poland; agata.przybys@gmail.com (A.P.-M.); izabella_antoniuk@sggw.edu.pl (I.A.); michal_kruk@sggw.edu.pl (M.K.); 2 Department of Mechanical Processing of Wood, Institute of Wood Sciences and Furniture, Warsaw University of Life Sciences, 02-776 Warsaw, Poland

**Keywords:** tool state monitoring, machine learning, milling chipboard

## Abstract

In this article, we present a novel approach to tool condition monitoring in the chipboard milling process using machine learning algorithms. The presented study aims to address the challenges of detecting tool wear and predicting tool failure in real time, which can significantly improve the efficiency and productivity of the manufacturing process. A combination of feature engineering and machine learning techniques was applied in order to analyze 11 signals generated during the milling process. The presented approach achieved high accuracy in detecting tool wear and predicting tool failure, outperforming traditional methods. The final findings demonstrate the potential of machine learning algorithms in improving tool condition monitoring in the manufacturing industry. This study contributes to the growing body of research on the application of artificial intelligence in industrial processes. In conclusion, the presented research highlights the importance of adopting innovative approaches to address the challenges of tool condition monitoring in the manufacturing industry. The final results provide valuable insights for practitioners and researchers in the field of industrial automation and machine learning.

## 1. Introduction

Using sensors in various stages of the furniture manufacturing process in order to evaluate its various stages is a common trend in the topic of automation-related research. The problem itself is complex, containing multiple steps that often require a high level of precision, and can require additional adjustments if even the smallest elements are added or exchanged. The introduction of advanced technology into these processes is innovative and helps to streamline them. This is particularly critical for tool condition monitoring, where incorrect or poorly timed decisions about replacement can lead to reduced product quality and subsequent loss for the manufacturing company [1,2,3,4].

One key focus of the research presented in this paper is the milling process, where any inaccurate decisions can be highly influential. The application of sensor-based technology to monitor tool conditions brings a fresh perspective to these problems. Checking the state of the tool, as in other stages, can be performed manually, but it is a time-consuming process that requires pausing the production. The automation of this process, therefore, represents a significant advancement in the field.

Tool monitoring in general is a widely discussed and evaluated topic [5,6,7]. It involves the gradual deterioration of the cutting edge, which results in decreasing product quality. It is important to note that any automatic solution should strive to avoid two situations: unnecessarily stopping the production process while the tool is still in good condition and delaying the exchange past the point when it is in bad enough condition to produce unacceptable products. Such a solution needs to be precise and provide some feedback in an automatic and online way. Using a specialized set of sensors focused on recording specific signals from the production line and evaluating data from these signals seems to be the best approach to that aspect [8,9].

A major innovation presented in this work is the way that sensor data are used to solve the complex problems inherent in tool condition monitoring. While furniture manufacturing can involve numerous materials, wood-based ones are the most common. The presented approach to data-driven tool condition monitoring opens up new possibilities for improving manufacturing processes in this industry. There are numerous works focusing on such elements [10,11]. Depending on the specific task, different signals are checked and evaluated, verifying how useful they can be in identifying tool condition during various stages of the machining process [3,12,13,14,15,16]. While the problems involved are well described, there still is a need for an automatic and precise solution that is easy to incorporate in production and possible to implement in actual work environments.

Due to the problem’s overall complexity, using machine learning algorithms seems to be the best option. Machine learning algorithms have become increasingly important in manufacturing processes, and the innovative approach proposed in this paper aims to apply these techniques to tool condition monitoring. Current research already includes various approaches, used both for image- and sensor-based systems [14,17,18,19,20]. The presented method extends these approaches, introducing novel ways of applying machine learning algorithms to tool condition monitoring tasks. Depending on the chosen approach, various problems, their aspects and potential applications of the proposed solutions are considered. Solutions such as the one used for tree species recognition, presented in [21], show that machine learning algorithms can be adapted even to the most complicated tasks if the appropriate input data and training process are used.

When it comes to the problem of tool condition monitoring specifically, the main division refers to the different parts used. While recording signals is a commonly used approach, some solutions consider using images, often paired with Convolutional Neural Networks (CNNs), which perform relatively well when such samples are considered [1,2,22,23,24]. Additionally, the training process can be improved by using transfer learning with various pretrained networks (such as AlexNet [25,26] prepared for ImageNet database [27,28]) or data augmentation.

While solutions using images are quite popular due to the simplicity of the input collection process, they are not without drawbacks. In order to achieve high accuracy levels, large amounts of uniform training data are necessary. They also require tight cooperation with the manufacturer in order to pinpoint the key factors that should be considered, while not all features influencing the product quality are easy or straightforward to derive. In that regard, signals are better solutions, since it is easier to measure any potential changes. One problem to consider is ensuring the proposed approach is able to compute large amounts of data obtained in such a way.

One major innovation of research presented in this paper is the way in which sensor-based data are handled. Incorporating sensor-based data in neural-network-based solutions can pose a series of problems. The presented innovative approach to these problems includes new ways of dealing with discrepancies and variations in sensor data, leading to more accurate and reliable solutions. First of all, while the changes in recorded signals will occur while the tool’s state is deteriorating, not all of them will be consistent throughout all sensors. Such discrepancy can lead to the final solution’s inability to point out when the tool reaches the problematic state and needs to be exchanged. The second problem relates to the size of files obtained from different sensors. For signals requiring a high level of precision during the recording process, the resulting data files will be much larger, resulting in very different sizes of individual inputs. Any sensor-based solution needs to use such data in an optimal way, while retaining the advantage given by more precise measurements.

Some approaches address this problem by transferring the measured signals into images. In [29], the authors transfer sound signals to images using Short-Time Fourier Transform. The original data are first denoised and later converted to images. A pretrained CNN model performs deep feature extraction [22]. In the final method step, the Support Vector Machine is used for classification. Another approach converts the signals to the scalograms [30]. Constant-Q Transform with Nonstationary Gabor Transform is used, fusing vibration and acoustic single features with a multi-input CNN solution. The goal is to diagnose the state of the induction motor, and the above methodology was chosen due to the fact that in the authors’ opinion, the Continuous Wavelet Transform (CWT) was too time-consuming. While such an approach is faster, the overall solution accuracy suffers because of it.

The main focus of the research presented in this paper is the practical application of the novel solution to the problem of tool state recognition with input data based on the physical parameters of the used machinery. It is important for the given solution to allow easy implementation in the work environment, with high overall accuracy. The unique approach to feature generation, using Short-Time Fourier Transform (STFT) and Discrete Wavelet Transform (DWT) methods, sets this work apart from previous studies. Different variants of the method were tested for all selected, state-of-the-art classifiers, achieving more than satisfactory results.

The current approaches to tool condition monitoring in the chipboard milling process can be broadly classified into two categories: rule-based and data-driven. Rule-based approaches rely on expert knowledge and heuristics to detect wear and predict tool life. These approaches are often based on simple threshold values and are limited by the accuracy of the expert knowledge. Data-driven approaches, on the other hand, use machine learning algorithms to learn from the data and make predictions. These approaches are more flexible and can adapt to changing conditions, but they require large amounts of data and may be limited by the quality of them.

This paper proposes a data-driven approach using a combination of feature extraction and machine learning algorithms. The authors use 11 signals to extract features related to tool wear and use these features to train many classifiers, which are then used to predict tool wear and tool life.

This article makes several new contributions to the field of tool condition monitoring in the milling chipboard process. Firstly, the use of acoustic emission signals for feature extraction is a novel approach that has not been widely used in the literature. Secondly, the combination of feature extraction and machine learning algorithms has been shown to be effective in improving the accuracy of tool wear detection and tool life prediction. Finally, this article provides a detailed analysis of the performance of the proposed methodology and compares it with the current state-of-the-art approaches. In conclusion, this article proposes a novel approach for tool condition monitoring using a combination of feature extraction and machine learning algorithms. This approach has been shown to be effective in detecting tool wear and predicting tool life, and a detailed analysis of its performance is provided. The new contributions of this article include the use of acoustic emission signals for feature extraction and the combination of feature extraction and machine learning algorithms.

## 2. Data Acquisition

In the presented research, the main goal consisted of building a diagnostic system capable of accurate measurement of tool wear level without the need to stop the production process. The evaluation is based on the collected set of signals. All tests and recordings were conducted using a Jet 130 CNC machining center (Busellato, Thiene, Italy) with single, 40 mm exchangeable edge cutter head with an exchangeable carbide cutting edge (Faba SA, Baboszewo, Poland).

A sample of a chipboard panel with dimensions of 300 × 150 mm was used for tests. The element was mounted on a measuring platform. A 6 mm deep groove was milled, with spindle speed set at 18,000 rpm and feed rate equal to 0.15 mm per tooth. The selection of these parameters was based on a thorough analysis of the literature and the authors’ own experience in chipboard milling. A rotational speed of 18,000 rpm was chosen, as it is a commonly used speed in the industry. A feed rate of 0.15 per tooth was selected, as it is a value that has been shown to provide good results in terms of surface finish and tool wear. A cutting depth of 6 mm was chosen, as it is a value that is commonly used in the industry for milling chipboard panels of this size.

**Figure 1:**
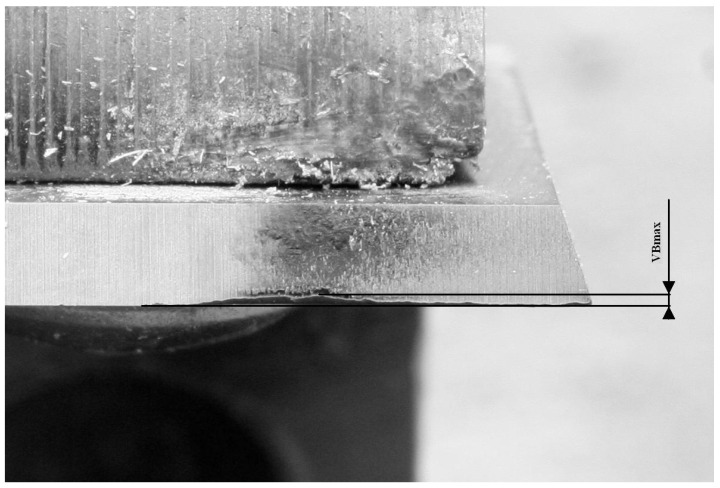
A microscopic photo of drill bit wear, with an outline of the VBmax parameter used for class evaluation.

Tool state was classified as one of these three states: Green, Yellow and Red. The first state refers to a new tool that remains in good condition. The Yellow state refers to an element in an intermediate state but is still usable. Finally, the Red class denotes tools that need to be exchanged due to their high wear level. In order to accurately denote each state, the VBmax parameter was used, as shown at Figure 1.

During each of the experiments, tasks were temporarily interrupted, and the current condition of the blade was subjected to physical measurements using a Mitutoyo TM-505 microscope. It is well suited for measuring dimensions and angles. Moreover, a Mitutoyo measuring microscope can be used to check the shape of screws and gears by attaching an optional reticle. Using this equipment, wear states were measured and assigned to one of the three wear states according to the following set of rules:

- If VBmax is in the range (0–0.15) mm, then it is a Green state—four different levels of wear state;
- If VBmax is in the range (0.151–0.299) mm, then it is a Yellow state—two different levels of wear state;
- If VBmax is in the range (>0.299) mm, then it is a Red state—two different levels of wear state.

The experimental system has multiple sensors with the ability to collect a total of 11 parameters, which are the following:

- Force value in the (1) *X* and (2) *Y* axes (Kistler 9601A sensor; Impexron GmbH, Pfullingen, Germany);
- (3) Acoustic emission (Kistler 8152B sensor; Kistler Group, Winterthur, Switzerland);
- (4) Noise level (Brüel & Kjær 4189 sensor; Brüel and Kjær, Nærum, Denmark);
- (5) Vibration level (Kistler 5127B sensor; Kistler Group, Winterthur, Switzerland);
- (6) Device-rated current (Finest HR 30 sensor; Micom Elektronika, Zagreb, Croatia);
- (7) Device-rated voltage (Testec TT-Si9001 sensor; Testec, Dreieich, Germany);
- (8) Head-rated current (Finest HR 30 sensor; Micom Elektronika, Zagreb, Croatia);
- (9) Head-rated voltage (Testec TT-Si9001 sensor; Testec, Dreieich, Germany);
- (10) Servo-rated current (Finest HR 30 sensor; Micom Elektronika, Zagreb, Croatia);
- (11) Servo-rated voltage (Testec TT-Si9001 sensor; Testec, Dreieich, Germany).

National Instruments PCI-6111 measurement cards (for measuring acoustic emissions) and PCI-6034E (for measuring other parameters) were used for data acquisition from the sensors.

The recording was carried out using a PC with National Instruments software, i.e., the Lab ViewTM (National Instruments Corporation, ver. 2015 SP1, Austin, TX, USA) environment using the NI PCI-6034E and NI PCI-6111 (Austin, TX, USA) data acquisition cards. In order to adequately record the AE signal, a card with high sampling frequency was necessary (2 MHz, measuring window of 0.3 s). For the remaining signals card with a frequency of 50 kHz, a 1.1 s measuring window was used. Each signal was connected to cards separately for each frequency range. BNC-2110 connection boxes were used for this task.

**Figure 2:**
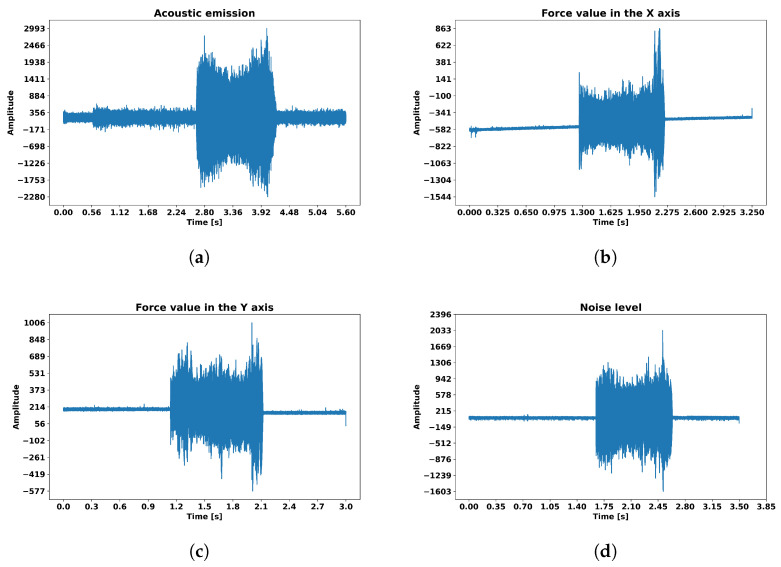
Example raw signals for (**a**) acoustic emission, (**b**) force X, (**c**) force Y and (**d**) noise level.

Since potential, irregular noises and changes in sound could influence the training process, all sensors were kept in the same position relative to the workpiece and cutting zone throughout the entire measurement process. The overall structure of the data collected during this stage is outlined in Table 1. Figure 2 shows plots with example raw signals for acoustic emission, force X, force Y and noise level.

## 3. Sensor Fusion

The presented approach to tool condition monitoring involves the use of multiple sensors to collect data on various physical parameters of the machinery. The collected data are then used to train machine learning algorithms to accurately predict the tool state.

In order to improve the accuracy of the performed predictions, a sensor fusion approach was applied, which combines the information from multiple sensors. Specifically, a feature-level fusion approach was used.

The feature extraction process involves applying various signal processing techniques to the raw sensor data to extract relevant features. For example, Short-Time Fourier Transform (STFT) and Discrete Wavelet Transform (DWT) methods are used to extract features from the acoustic emission signal. Similarly, statistical methods are used to extract features from all the signals mentioned in the previous section. Once the features are extracted from each sensor, they are combined into a single feature vector using a concatenation operation. The resulting feature vector is then used as input to the machine learning algorithms.

Various machine learning algorithms were tested, including K-Nearest Neighbors, GaussianNB, MultinomialNB, Stochastic Gradient Descent, Decision Tree, Random Forest, Gradient Boosting, Extreme Gradient Boosting, Light Gradient Boosting and Support Vector Machine, as described in Section 4.6. The main finding in the performed tests pointed out that the best results were obtained using a combination of features derived from all of signals (sensors). The presented approach improves the accuracy of final predictions and allows more effective tool state monitoring during the chipboard milling process.

## 4. Methods

After collecting the initial signals, additional preparation was required in order to prepare them for later usage in AI methods. In the current approach, two different methods were considered for the sample splitting: Short-Time Fourier Transform (STFT) and Discrete Wavelet Transform (DWT). In order to analyze the effectiveness of chosen approaches, a set of state-of-the-art classifiers was chosen.

### 4.1. Measuring Apparatus and Parameters

In durability tests, the condition of the cutting tools was assessed. For this purpose, the VBmax indicator was used (maximum wear on the flank surface—Figure 1). VBmax was read on a MitutoyoTM-505 instrument microscope.

**Figure 3:**
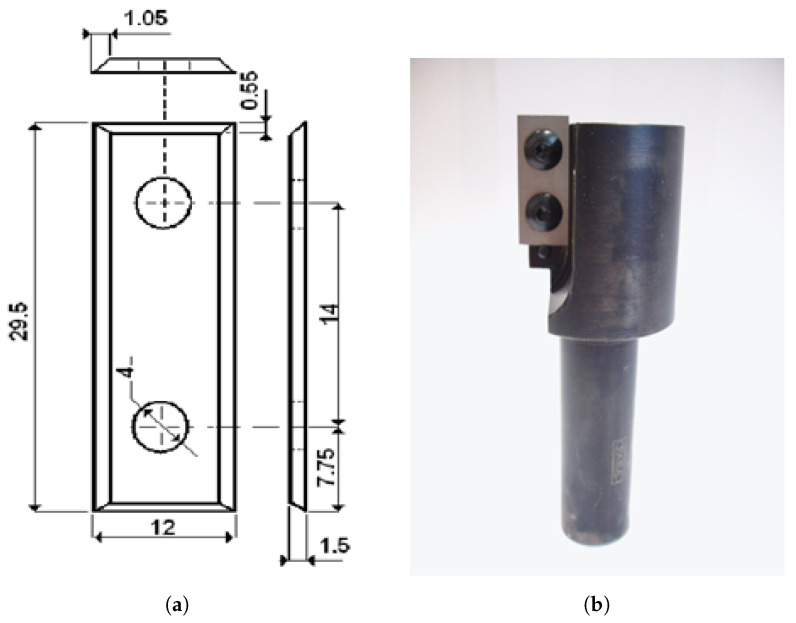
The geometry of the replaceable cutter for the milling head (**a**) and general view of the milling head (**b**).

**Figure 4:**
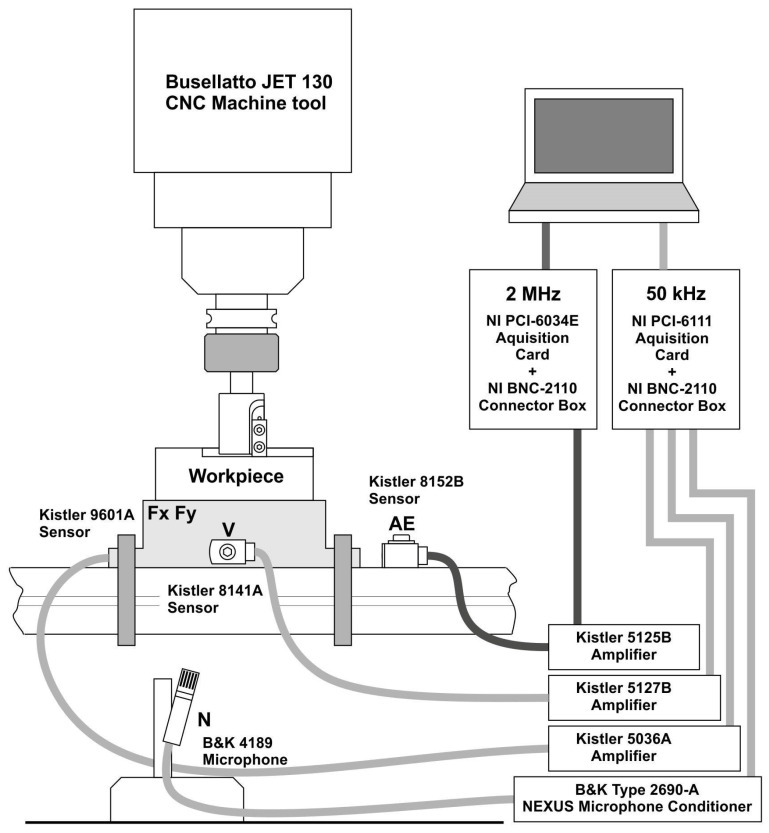
Scheme of the test stand layout used during the experiments.

The main part of the research was carried out on the Busselato Jet 130 industrial machining center, which is part of the equipment of the Machine Tools and Wood Processing Department of the Warsaw University of Life Sciences (SGGW). The machine tool is equipped with a Faba single-edged milling head (Figure 3a) with a diameter of 40 mm and a replaceable blade; the geometry is shown in Figure 3b. The standard blades are made of sintered carbide, with the symbol KCR08. The overall parameters of the sintered carbide and 50HS spring steel are presented in Table 2 and Table 3. The standards used during the experiments are presented in Table 4. The full list of used equipment is shown in Table 5, while Figure 4 outlines the test stand setup.

### 4.2. Data Transformation

Before any additional operations were performed, data normalization was required to ensure that the discrepancies in the data size for different signals would not influence the training process. Preprocessing is an essential step in the machine learning pipeline, as it ensures that the data are appropriately prepared and transformed for the chosen algorithms. Two prevalent techniques used for this purpose are normalization and standardization.

Normalization is a technique that scales the data into a specific range, typically between 0 and 1, or sometimes −1 and 1. The purpose of normalization is to bring all features to the same scale and prevent any feature from dominating the model due to its original scale. The most common method for normalization is MINMAX scaling.

Standardization, on the other hand, transforms the data such that they have a mean of 0 and a standard deviation of 1. The purpose of standardization is to make the data comparable across different features by removing the effects of various units or scales.

The main differences between normalization and standardization are as follows:

- **Range**: Normalization scales the data to a specific range (usually between 0 and 1), whereas standardization scales the data to have a mean of 0 and a standard deviation of 1.
- **Robustness**: Normalization is sensitive to outliers, as the scaling is directly dependent on the minimum and maximum values. In contrast, standardization is more robust to outliers, as it uses the mean and standard deviation, which are less influenced by extreme values.
- **Use Cases**: Normalization is preferred when the algorithm is sensitive to the scale of the input features, such as in neural networks or K-Nearest Neighbors. Standardization is more suitable for linear models, such as logistic regression or Support Vector Machines, which assume that the input features are normally distributed.

In summary, both normalization and standardization are essential preprocessing techniques in machine learning, with distinct purposes and use cases. The choice between these techniques depends on the specific requirements of the algorithm and specifics of the data being used.

#### 4.2.1. Data Normalization

In the presented approach, the MinMaxScaler estimator was used, since it is one of the most commonly used algorithms for this aspect. In this method, each feature is scaled and translated individually, translating original values for the training set in the given range (i.e., so that all values fit between the zero and one range). This operation was calculated as shown in Equation (1):

(1) $${\tilde{x}}_{i}=\frac{x_{i}-x_{min}}{x_{max}-x_{min}}$$

#### 4.2.2. Data Standardization

In machine learning, data standardization is a common preprocessing step to ensure that features are on a similar scale, thus helping the model to converge faster and perform better. A widely used method for data standardization is the Z-score normalization, which can be defined as

(2) $$z=\frac{x-\mu }{\sigma }$$

where *z* is the standardized value, *x* is the original value, μ is the mean of the feature and σ is the standard deviation of the feature. This process is applied to each feature independently, transforming the data such that they have a mean of 0 and a standard deviation of 1. Standardizing the data can be particularly helpful in algorithms that are sensitive to feature scales, such as gradient- or distance-based methods.

### 4.3. Short-Time Fourier Transform

In the presented experiments, the first method used for the sample splitting process was STFT. In this operation, a 32-segment version of the method was used in order to split initial samples by their frequency. It was repeated for all 11 recorded signals, based on the sampling frequency for each of them (see Table 1). The no-overlap parameter was omitted in order to minimize data duplication—the transform did not include the overlapping windows. The range was defined using the Hamming window, and due to system symmetry, only half of the segments (or bins) were used for calculation: (32/2) + 1, giving a total of 17 segments.

The Short-Time Fourier Transform (STFT) is a widely used technique for analyzing the time–frequency content of a signal. It works by partitioning the signal into overlapping segments, applying a window function and then computing the discrete Fourier transform of each segment. This allows the extraction of localized frequency information, which can be useful for a variety of applications, such as speech processing, audio analysis and signal processing.

The algorithm presented here computes a set of features from a given signal using the STFT method. The main function, CalcFeatures_stft, takes as input a signal *x*, a custom sampling frequency fs_custom and optional parameters for the window function (window), the number of data points per segment (nperseg) and the number of overlapping points between segments (noverlap). The default values for these parameters are set as follows: window=‘hamming’, nperseg=32, and noverlap=0.

The algorithm first performs the STFT on the input signal using the specified parameters, obtaining the time–frequency representation Zxx. The absolute values of the complex coefficients are then calculated and stored in the matrix *z*. For each frequency bin (i.e., each row of *z*), the algorithm computes a set of statistical features:

1. The mean value (y1).
2. The maximum value (y2).
3. The root-mean-square value (y3).
4. The standard deviation (y4).
5. The coefficient of variation, computed as the standard deviation divided by the mean (y5).
6. The ratio of the maximum value to the mean value (y6).

Finally, the features are concatenated into a single vector and returned as the output.

After the above calculations, the final set contained total of 102 variables (17 × 6) for each of the signal subsets. The complete set of variables for all of the used signals therefore contained 11 (signals) × 102 = 1122 variables.

By analyzing the time–frequency content of the signal and extracting statistical features, this algorithm can provide valuable information for further analysis or machine learning tasks. The use of STFT makes it particularly well suited for applications where the signal’s frequency content varies over time, such as in audio and speech processing. The algorithm overview is presented in Algorithm 1.
**Algorithm 1** CalcFeatures_stft function1:**procedure** CalcFeatures_stft(x,fs_custom,window=‘hamming’,nperseg=32,noverlap=0)2:    (f,t,Zxx)←STFT(x,fs=fs_custom,window=window,nperseg=nperseg,noverlap=noverlap)3:    z←abs(Zxx)4:    n←numberofrows(z)5:    m←numberofcolumns(z) 6:    Calculate statistical features for each row of *z*:
y1←mean(z,axis=1)y2←max(z,axis=1)y3←RMS(z,axis=1)y4←std(z,axis=1)y5←std(z,axis=1)/mean(z,axis=1)y6←max(z,axis=1)/mean(z,axis=1) 7:    features←concatenate(y1,y2,y3,y4,y5,y6,axis=0)8:    **return** features9:**end procedure**

### 4.4. Discrete Wavelet Transform

The second method used was Discrete Wavelet Transform (DWT). DWT is a powerful signal processing technique that allows efficient multiresolution analysis of a given signal. It decomposes it into a set of wavelet coefficients, which can capture both the frequency and time information simultaneously. DWT operates by iteratively breaking down a signal into two parts: approximation coefficients (low-frequency components) and detail coefficients (high-frequency components).

The core idea behind DWT is to use a pair of complementary functions, called mother wavelet and scaling function. The mother wavelet is used to analyze high-frequency details in the signal, while the scaling function is responsible for capturing the low-frequency, or smooth, aspects of it. The wavelet decomposition is achieved by convolving the signal with these two functions and then downsampling the result by a factor of 2 at each level.

The process of wavelet decomposition is applied recursively to the approximation coefficients, resulting in a multilevel decomposition. At each level, the signal is further analyzed, and additional detail coefficients are extracted, representing various frequency bands. This hierarchical structure enables the preservation of the signal’s temporal and frequency characteristics across different scales, making it suitable for a wide range of applications, including compression, denoising and feature extraction.

In the presented implementation, DWT is performed using the specified wavelet (sym5) and iterating through the given decomposition levels (7 in this case). At each level, the function calculates several statistical features from both the approximation and detail coefficients. These features capture essential information about the signal and can be used for further analysis or as an input for the chosen AI model.

By utilizing DWT in this function, one can take advantage of the time–frequency localization properties of wavelet analysis to derive meaningful features from the input signal, which can help enhance the performance of AI models in various applications.

The CalcFeatures_cwt function aims to extract a set of features from an input signal using Discrete Wavelet Transform (DWT) for multiresolution analysis. The function accepts three parameters: the input signal *x*, the wavelet name (in this case, ‘sym5’), and the number of decomposition levels (set to 7). The ‘sym5’ wavelet, also known as the Symlet 5 wavelet, is part of the Symlet wavelet family, known for its near symmetry and good frequency response.

The function begins by initializing an empty list called feature_levels to store the feature sets calculated at each decomposition level. It then iterates through the specified number of levels, performing the following steps:

1. **Wavelet decomposition**: At each level, the input signal (or the approximation coefficients from the previous level) is decomposed using the DWT with the specified wavelet. This produces two sets of coefficients: approximation coefficients (*x*) and detail coefficients (coeff_d).
2. **Feature extraction for approximation coefficients**: The function calculates six statistical features from the approximation coefficients (*x*) that describe the signal’s low-frequency components. These features include the mean, maximum, root mean square (RMS), standard deviation, coefficient of variation (standard deviation divided by mean) and the ratio of the maximum value to the mean value.
3. **Feature extraction for detail coefficients**: The function calculates ten statistical features from the detail coefficients (coeff_d) that represent the high-frequency components, or noise, in the signal. These features include the mean, maximum, RMS, standard deviation, coefficient of variation, ratio of the maximum value to the mean value and the 5th, 25th, 75th and 95th percentiles.
4. **Feature set construction**: The 16 features derived from the approximation and detail coefficients are combined into a single tuple. This tuple represents the feature set for the current decomposition level.
5. **Storing feature sets**: The feature set tuple is appended to the feature_levels list, which collects the feature sets for each decomposition level.

After iterating through all decomposition levels, the function returns the feature_levels list containing the feature sets for each level. These feature sets can be used for further analysis, such as signal classification, anomaly detection, or as input for an AI model to improve its performance in various applications.

The CalcFeatures_cwt function leverages the time–frequency localization properties of DWT to efficiently extract meaningful features from the input signal, making it a versatile and valuable tool for a wide range of signal processing tasks. The comprehensive outline of this function is presented in Algorithm 2.
**Algorithm 2** CalcFeatures_cwt function  1:**procedure** CalcFeatures_cwt(x,waveletname,level)  2:    feature_levels←emptylist  3:    **for** ii∈{0,1,…,level−1} **do**  4:        (x,coeff_d)←DWT(x,waveletname)  5:        Calculate statistical features for *x*:
wave_y1←mean(x)wave_y2←max(x)wave_y3←RMS(x)wave_y4←std(x)wave_y5←std(x)/mean(x)wave_y6←max(x)/mean(x)  6:        Calculate statistical features for coeff_d:
wave_coef_y1←mean(coeff_d)wave_coef_y2←max(coeff_d)wave_coef_y3←RMS(coeff_d)wave_coef_y4←std(coeff_d)wave_coef_y5←std(coeff_d)/mean(coeff_d)wave_coef_y6←max(coeff_d)/mean(coeff_d)wave_coef_n5←5%−percentile(coeff_d)wave_coef_n25←25%−percentile(coeff_d)wave_coef_n75←75%−percentile(coeff_d)wave_coef_n95←95%−percentile(coeff_d)  7:         features←(wave_y1,wave_y2,wave_y3,wave_y4,wave_y5,wave_y6,               wave_coef_y1,wave_coef_y2,wave_coef_y3,wave_coef_y4,               wave_coef_y5,wave_coef_y6,wave_coef_n5,wave_coef_n25,wave_coef_n75,               wave_coef_n95)  8:        Append features to feature_levels  9:    **end for**10:    output←Flatten(feature_levels)11:    **return** output12:**end procedure**

### 4.5. Hyperparameter Optimization for Classifiers

Hyperparameter optimization plays a crucial role in enhancing the performance of machine learning models. This paper discusses the scientific method of employing an exhaustive grid search for hyperparameter optimization, thereby improving the accuracy and efficiency of various machine learning algorithms tested during the experiments.

Machine learning model performance relies heavily on selecting the appropriate hyperparameters—adjustable parameters that control the learning process of a model. An exhaustive grid search is a widely used technique that explores a specified parameter space to find the optimal combination of hyperparameters for a given model.

To perform hyperparameter optimization using an exhaustive grid search, the following steps are undertaken:

1. **Import necessary libraries:** First, import the required libraries that provide the functions and algorithms for implementing the exhaustive grid search.
2. **Load and preprocess the dataset:** Load the dataset and perform necessary preprocessing tasks, such as feature engineering, data cleaning and splitting into training and testing sets.
3. **Define the model:** Choose the desired machine learning algorithm (e.g., Random Forest Classifier, Support Vector Machines, etc.) and instantiate the model with default or initial hyperparameters.
4. **Specify the hyperparameter grid:** Define a dictionary or a structured data format containing the hyperparameters and their respective ranges to be explored during the grid search. For example:
  - “n_estimators”: [10, 50, 100, 200],
  - “max_depth”: [None, 10, 20, 30],
  - “min_samples_split”: [2, 5, 10],
  - “min_samples_leaf”: [1, 2, 4],
  - “bootstrap”: [True, False].
5. **Initialize the grid search:** Instantiate an exhaustive grid search algorithm with the chosen model, hyperparameter grid, scoring metric and cross-validation strategy.
6. **Fit the model:** Train the model using the training data while performing an exhaustive search for the best hyperparameters.
7. **Extract optimal hyperparameters:** Retrieve the best combination of hyperparameters found during the search.
8. **Evaluate model performance:** Assess the performance of the model with the optimal hyperparameters on the test dataset and compare it with the baseline model.

Employing an exhaustive grid search for hyperparameter optimization allows for the identification of the best hyperparameter combination, resulting in improved model performance and accuracy. Although this method can be computationally expensive due to its exhaustive search approach, the benefits of optimizing hyperparameters can significantly enhance the overall effectiveness of machine learning models. The full overview is presented in Algorithm 3.
**Algorithm 3** Exhaustive Grid Search for Hyperparameter Optimization  1:**procedure** GridSearch(model,param_grid,dataset,scoring,cv)  2:    Preprocess dataset  3:    Split dataset into train_set and test_set  4:    Initialize best_score←−∞  5:    Initialize best_params←∅  6:    **for all** combinations params in param_grid **do**  7:          model← Instantiate model with params  8:          scores← Perform cv-fold cross-validation on train_set with model  9:          avg_score← Average scores10:          **if** avg_score>best_score **then**11:               best_score←avg_score12:               best_params←params13:          **end if**14:    **end for**15:    Train model with best_params on entire train_set16:    Evaluate model on test_set using scoring metric17:    **return** model, best_params18:**end procedure**

### 4.6. Classifiers

The main goal for the chosen classifier set was to evaluate previously prepared variables, checking the status to which each example will be qualified. In order to analyze and evaluate the overall accuracy, as well as verify the obtained results, 10 state-of-the-art classifiers were chosen and implemented, testing both variable sets obtained using the STFT and DWT approaches.

#### 4.6.1. K-Nearest Neighbors

The first chosen classifier was K-NN, since it is often described as one of the most important, non-parametric classification methods [31,32]. It assigns the object class based on its neighborhood, checking to which of the available classes most of the current neighbors belong. If, in some cases, the highest number of neighbors is identical for more than one class, the final classification will be decided based on distances to each of them.

The standard version of the algorithm calculates Euclidean distance, and it is not often used. Various improvements to this method were made, one of them incorporating the Neighborhood Components Analysis (NCA). It is used to maximize a stochastic variant of the leave-one-out K-NN scores on the training set, maximizing the sum over all available samples of the probability that the current sample is correctly classified. In the approach presented in this paper, the K-NN method used was configured using following parameters:

- ‘kneighborsclassifier__n_neighbors’: [3, 4, 5, 6, 7, 8, 9, 10, 11, 12, 13, 14, 15, 16, 17, 18, 19, 20];
- ‘kneighborsclassifier__weights’: [‘uniform’, ‘distance’];
- ‘kneighborsclassifier__metric’: [‘minkowski’, ‘euclidean’, ‘manhattan’];

#### 4.6.2. GaussianNB

The original version of the Naive Bayesian Classifier is based on the Bayes’ theorem, with the assumption of conditional independence between pairs of features given the value of the class.

The assumption of Bayes’ theorem is the following relationship [33,34]:

(3) $$P\left(y|x_{1},\ldots ,x_{n}\right)=\frac{P\left(y\right)P\left(x_{1},\ldots ,x_{n}|y\right)}{P(x_{1},\ldots ,x_{n})}$$

where y is a class variable, and $x_{i}$ consists of the dependent feature vector, including the naive conditional independence assumption:

(4) $$P\left(x_{i}|y,x_{1},\ldots ,x_{i-1},x_{i+1},\ldots ,x_{n}\right)=P\left(x_{i}|y\right)$$

additionally assuming that the likelihood of the features is Gaussian:

(5) $$P\left(x_{i}|y\right)=\frac{1}{\sqrt{2\pi \sigma_{y}^{2}}}\exp\left(-\frac{{\left(x_{i}-\mu_{y}\right)}^{2}}{2\sigma_{y}^{2}}\right)$$

where the parameters $\sigma_{y}$ and $\mu_{y}$ are estimated using maximum likelihood.

The final parameters used for this approach are ‘var_smoothing’: np.logspace(0, −9, num = 100).

#### 4.6.3. MultinomialNB

The Multinomial Naive Bayesian Classifier is also based on the Bayes’ theorem but additionally includes multinomially distributed data [35,36]. In this approach, vectors $\theta_{y}=\left(\theta_{y1},\ldots ,\theta_{yn}\right)$ generate the multinominal distribution for each class y, where $\theta_{yi}$ is the probability of feature i appearing in a sample belonging to class y, and n is the number of features used.

The parameters $\theta_{y}$ are calculated as follows:

(6) $${\hat{\theta }}_{yi}=\frac{N_{yi}+\alpha }{N_{y}+\alpha n}$$

where $N_{yi}=\sum_{x\in T}x_{i}$ is the number of times feature i appears in a sample of class y in the training set *T*, and $N_{y}=\sum_{i=1}^{n}N_{yi}$ is the total count of all features for class y.

The parameters for the MultinominalNB method are as follows: ‘alpha’: [1, 0.1, 0.01, 0.001, 0.0001, 0.00001].

#### 4.6.4. Stochastic Gradient Descent

Another iterative method used to optimize the overall solution, as well as its classification, was Stochastic Gradient Descent. It is is based on the Robbins–Monro algorithm [37], with the main goal being a stochastic approximation for the given set optimization. In order to achieve this, the set is estimated randomly from a given subset. This solution is computationally efficient but not accurate in the convergence criterion [38,39].

The main goal is to learn a linear scoring function $f\left(x\right)=w^{T}x+b$ with the model parameters $w\in R^{m}$ and intercept b∈R, while minimizing the regularized training error, denoted as

(7) $$E\left(w,b\right)=\frac{1}{n}\sum_{i=1}^{n}L\left(y_{i},f\left(x_{i}\right)\right)+\alpha R\left(w\right)$$

where *L* is the loss function, *R* defines the regularization term that penalizes model complexity and α>0 is a non-negative hyperparameter that controls the regularization strength. The loss function is defined as

(8) $$L\left(y_{i},f\left(x_{i}\right)\right)=\max\left(0,1-y_{i}f\left(x_{i}\right)\right)$$

and the used regularization term is denoted as

(9) $$R\left(w\right)=\frac{1}{2}\sum_{j=1}^{m}w_{j}^{2}={\left||w|\right|}_{2}^{2}$$

The main application of the core version of SGD is unconstrained optimization problems. It can approximate the true gradient of E(w,b) by considering a single training example at a time. For each example, the model parameters are updated as follows:

(10) $$w\leftarrow w-\eta \left[\alpha \frac{\partial R(w)}{\partial w}+\frac{\partial L(w^{T}x_{i}+b,y_{i})}{\partial w}\right]$$

where η is the learning rate and *b* denotes the intercept parameter.

The presented implementation used Stochastic Gradient Descent with the following parameters:

- ‘sgdclassifier__loss’: [‘log_loss’];
- ‘sgdclassifier__penalty’: [‘elasticnet’, ‘none’];
- ‘sgdclassifier__alpha’: np.logspace (−5, 5, 10).

#### 4.6.5. Decision Tree

When it comes to the decision criteria, Decision Trees are the simplest and most popular classifiers used [40]. In this case, the base for the decision-making process is narrowing down the results by range classes. At the same time, this algorithm might require larger training sets to achieve a satisfactory accuracy rate [41].

In this algorithm, the feature space is recursively partitioned so that samples with the same labels are grouped together.

It is assumed that $x_{i}\in R^{n}$ are the training vectors, $y\in R^{l}$ is a label vector and *m* is the number of the node with data at node *m*, denoted as $Q_{m}$, and the number of samples at node *m*, indicated as $n_{m}$.

For each candidate split $\theta =(j,t_{m})$ consisting of a feature *j* and threshold $t_{m}$, the data are split into $Q_{m}^{left}\left(\theta \right)$ and $Q_{m}^{right}\left(\theta \right)$ subsets:

(11) $$\begin{array}{c} Q_{m}^{left}\left(\theta \right)=\left\{\left(x,y\right)|x_{j}\leq t_{m}\right\}\\ Q_{m}^{right}\left(\theta \right)=Q_{m}\setminus Q_{m}^{left}\left(\theta \right) \end{array}$$

Then, the decision about the node chosen to be the split is made according to

(12) $$G\left(Q_{m},\theta \right)=\frac{n_{m}^{left}}{n_{m}}H\left(Q_{m}^{left}\left(\theta \right)\right)+\frac{n_{m}^{right}}{n_{m}}H\left(Q_{m}^{right}\left(\theta \right)\right)$$

where H() is the loss function, very often as Gini:

(13) $$H\left(Q_{m}\right)=\sum_{k}p_{mk}\left(1-p_{mk}\right)$$

(14) $$p_{mk}=\frac{1}{n_{m}}\sum_{y\in Q_{m}}I\left(y=k\right)$$

where *k* is the number of classes, and *m* denotes the number of nodes.

Finally, it is recursively computed for subsets $Q_{m}^{left}\left(\theta^{*}\right)$ until the maximum depth is reached, $n_{m}<\min_{samples}$ or $n_{m}=1$.

In the presented calculations, the Decision Tree has the following parameters:

- ‘max_depth’: range (2, 10, 1);
- ‘criterion’: [‘gini’].

#### 4.6.6. Random Forest

Random Forest is an ensemble method, where the main idea is to combine the predictions of several base classifiers in order to improve robustness [42].

In this case, each tree in the ensemble is built from a sample drawn with replacement from the training set, with the best split being found also from a random subset of features [43,44].

An individual decision tree classifier has high variance and is prone to overfitting, which is not the case with the Random Forest classifier. Moreover, due to randomness, some errors can cancel out.The variance reduction often leads to a better model as a result.

The used Random Forest implementation has the following parameters:

- ‘n_estimators’: range (100, 1000, 100);
- ‘max_depth’: range (2, 10, 1);
- ‘bootstrap’: [True].

#### 4.6.7. Gradient Boosting

The next chosen algorithm is Gradient Boosting. It is a method that uses dependencies in the previous steps of the result prediction [45,46]. This algorithm often is the starting point for other improved methods [47,48].

The model itself is additive, where prediction ${\hat{y}}_{i}$ for a given features $x_{i}$ is based on

(15) $${\hat{y}}_{i}=F_{M}\left(x_{i}\right)=\sum_{m=1}^{M}h_{m}\left(x_{i}\right)$$

where $h_{m}$ denotes weak learners and M is the number of weak learners, followed with the greedy property of the method:

(16) $$F_{m}\left(x\right)=F_{m-1}\left(x\right)+h_{m}\left(x\right)$$

where $h_{m}$ minimizes a sum of losses $L_{m}$ from the previous ensemble $F_{m-1}$:

(17) $$h_{m}=\arg\min_{h}L_{m}=\arg\min_{h}\sum_{i=1}^{n}l\left(y_{i},F_{m-1}\left(x_{i}\right)+h\left(x_{i}\right)\right),$$

where $l(y_{i},F\left(x_{i}\right))$ is the loss function.

The mapping from the value $F_{M}\left(x_{i}\right)$ to a class is loss-dependent. For the log-loss, the probability that $x_{i}$ belongs to the positive class is denoted as follows:

(18) $$p\left(y_{i}=1|x_{i}\right)=\sigma \left(F_{M}\left(x_{i}\right)\right)$$

where σ is the sigmoid function.

In a case where multiple classes are considered, K trees (K classes) are built at each of the *M* iterations. The probability that $x_{i}$ belongs to class k is calculated using the softmax of the $F_{M,k}\left(x_{i}\right)$ values.

For the presented approach, the Gradient Boosting algorithm uses the following parameters:

- ‘max_depth’: range (2, 20, 1);
- ‘learning_rate’: [0.01, 0.05, 0.1];
- ‘subsample’: [0.5, 0.75, 1.00].

#### 4.6.8. Extreme Gradient Boosting

Extreme Gradient Boosting or XGBoost is an improved version of the initial solution [46,48,49,50]. It has many advantages in comparison with the standard Gradient Boosting method, including elements such as [51]:

- Regularization rules;
- Parallel processing;
- Built-in feature to handle missing values;
- Built-in cross-validation technique;
- Tree pruning feature.

In the presented approach, Extreme Gradient Boosting has the following parameters:

- ‘max_depth’: range (2, 10, 1);
- ‘n_estimators’: range (100, 1000, 100);
- ‘learning_rate’: [0.01, 0.05, 0.2, 0.3];
- ‘sampling_method’: [‘uniform’, ‘subsample’, ‘gradient_based’].

#### 4.6.9. Light Gradient Boosting

Among the classifiers used, another solution using the Gradient Boosting approach was also selected. LGBM, unlike the algorithms based on random trees, does not rely on sorting when finding the best split point. It is based on the decision histogram, providing the possibility to follow the path of the expected least loss in time [52,53].

In comparison with XGBoost, LGBM has vertical, leafwise growth, resulting in more loss reduction and higher accuracy.

Light Gradient Boosting has the following parameters:

- ‘n_estimators’: range (100, 1000, 100);
- ‘max_depth’: range (5, 30, 5);
- ‘learning_rate’: [0.01, 0.05, 0.1];
- ‘bagging_fraction’: [0.75];
- ‘num_leaves’: range (5, 30, 5);
- ‘min_data_in_leaf’: range (5, 30, 5).

#### 4.6.10. Support Vector Machine

The Support Vector Machine is a classification method [54,55] based on correctly mapping data to multidimensional space. To achieve this, a function separating these data is applied, declaring decision classes and building a hyperplane or set of hyperplanes in a high-dimensional space based on kernel functions. The main goal is the maximization of the separation margin—the largest distance to the nearest training data points of any class (also called support vectors) [56].

The margin is maximized by minimizing ${\left||w|\right|}^{2}=w^{T}w$, while penalties are given when a sample is misclassified:

(19) $$\begin{array}{c} \min_{w,b,\zeta }\frac{1}{2}w^{T}w+C\sum_{i=1}^{n}\zeta_{i}\\ \begin{array}{cc} \mathrm{subject}\mathrm{to} & y_{i}\left(w^{T}\phi \left(x_{i}\right)+b\right)\geq 1-\zeta_{i},\\ \; & \zeta_{i}\geq 0,i=1,...,n \end{array} \end{array}$$

where *C* is the penalty term which controls the penalty strength and $\zeta_{i}$ is the samples’ distance from their correct margin boundary.

The main problem can be changed to a dual problem:

(20) $$\begin{array}{c} \min_{\alpha }\frac{1}{2}\alpha^{T}Q\alpha -e^{T}\alpha \\ \begin{array}{cc} \mathrm{subject}\mathrm{to} & y^{T}\alpha =0\\ \; & 0\leq \alpha_{i}\leq C,i=1,...,n \end{array} \end{array}$$

where $\alpha_{i}$ is the dual coefficients, *e* is the vector of all ones, and by positive semidefinite matrix, $Q_{ij}=y_{i}y_{j}K\left(x_{i},x_{j}\right)$, and $K\left(x_{i},x_{j}\right)=\phi {\left(x_{i}\right)}^{T}\phi \left(x_{j}\right)$ is the kernel.

When multiple classes are considered, the “one-versus-one” approach is usually applied, meaning that m ∗ (m − 1)/2 classifiers are constructed, where m is number of classes.

In the presented approach, SVM has the following parameters:

- ‘svc__C’: [100, 200, 300, 400, 500, 600, 700, 800, 900, 1000, 3000, 5000, 10,000];
- ‘svc__gamma’: [0.001, 0.01, 0.1, 0.2, 0.3];
- ‘svc__kernel’: [‘rbf’];
- cv_set = StratifiedKFold(n_splits = 3, shuffle = True, random_state = 423).

### 4.7. General Implementation

All experiments were performed on hardware with the following specifications with an Ubuntu system:

- Processor: AMD RYZEN THREADRIPPER 2990WX (32C 64T) 4.3 GHz.
- Motherboard: AsRock X399 TAICHI.
- Memory: 8 × ADATA XPG SPECTRIX DDR4 16 GB D41 3000 MHz (128 GB RAM).
- Graphics Card: 2 × Nvidia GeForce RTX Titan 24 GB GDDR6 (48 GB RAM).
- Drive SSD: 2 × WD BLACK 1TB WDS100T3X0C1TB (PCIE).
- Drive HDD: 1 × WD RED PRO 8TB WD8003FFBX 3.5″ (SATA).
- Power supply: BE QUIET! DARK POWER PRO 11 1000 W.
- Cooling: BE QUIET! Silent Loop BW003 280 mm.
- Network: 10GbE SFP+.

### 4.8. Results of Numerical Experiments

In total, 72 variants of numerical experiments were performed. These variants consist of three data transformation methods and six combinations of data splitting into training and testing sets, as well as four different approaches. Cross-validation was used in all numerical experiments, randomly selecting the training and testing subsets 10 times. Specifically, the following variants were used:

1. Types of input data transformations:
  - Without data transformation (no standardization or normalization).
  - With standardization.
  - With MINMAX normalization.
2. Data splits for training/testing sets for cross-validation:
  - 20/80.
  - 30/70.
  - 40/60.
  - 50/50.
  - 60/40.
  - 70/30.
3. Four used approaches:
  - STFT approach without hyperparameters optimization.
  - STFT approach with hyperparameters optimization.
  - Wavelet approach without hyperparameters optimization.
  - Wavelet approach with hyperparameters optimization.

It was assumed that in order for the solution to be viable, especially due to the additional computation required for the exhaustive grid search during the hyperparameter optimization, it needed to reach a satisfactory accuracy rate. This threshold was set at 80%, and only results reaching this score are presented. For the results to be easily comparable between different approaches, each row in the tables displays the following information:

- The model’s performance with the specified transformation (denoted as **Transformation**);
- Number of training samples (**n_train**);
- Number of test samples (**n_test**);
- Percentage of data used for training (**% for training**);
- The model’s accuracy (**Acc. [%]**).

All the results are sorted in descending order of model accuracy.

The main goal of the performed experiments was to evaluate a range of machine learning models and data transformation techniques in order to understand their impact on classification accuracy. The models evaluated include Decision Trees (DT), Random Forests (RF), XGBoost Classifier (XGBC), Gradient Boosting (GB), K-Nearest Neighbors (KNN), Support Vector Machines (SVC), Stochastic Gradient Descent Classifier (SGDC) and Light Gradient Boosting Machine (LGBM). The dataset is transformed using MINMAX scaling (MINMAX), standard scaling (STD), and no scaling (NONE). Additionally, the experiments employ different train-test split ratios to examine their influence on model performance.

#### 4.8.1. Numerical Experiments for the STFT Approach, without Hyperparameters Optimization

The Table 6 presents the top results of numerical experiments conducted for the Short-Time Fourier Transform approach, without hyperparameter optimization. Various machine learning models are listed, each evaluated using three different transformations: MINMAX, STD, and NONE.

The table shows that Decision Trees achieved the highest accuracy (100%) when trained on 20% of the data (n_train = 60, n_test = 15), regardless of the transformation used. Meanwhile, Random Forests and Extreme Gradient Boosting achieved comparable performance when trained on 50% and 70% of the data, respectively. Gradient Boosting models demonstrated slightly lower accuracy scores, ranging between 83.02% and 86.67%.

Upon detailed analysis of the results, several observations can be made:

1. Decision Tree (DT) models with all three data transformation techniques consistently demonstrate perfect accuracy (100%) when using a 20% train–test split ratio.
2. Random Forest (RF) models exhibit high accuracy, with the best performance achieved using standard scaling and a 40% train–test split ratio, resulting in 98.33% accuracy.
3. XGBoost Classifier (XGBC) and Gradient Boosting (GB) models show variable accuracy levels, ranging from 66.67% to 95%. The best performance for XGBC is obtained using MINMAX scaling with a 40% train–test split ratio, while the highest accuracy for GB is achieved using standard scaling and a 60% train–test split ratio.
4. K-Nearest Neighbors (KNN) and Support Vector Machine (SVC) models demonstrate generally high accuracy, with KNN models reaching their peak performance using MINMAX scaling and a 20% train–test split ratio and SVC models performing the best using standard scaling and a 40% train–test split ratio.
5. Stochastic Gradient Descent Classifier (SGDC) models display moderate accuracy, ranging from 60% to 83.33%. The best performance is achieved using standard scaling and a 20% train–test split ratio.
6. Light Gradient Boosting Machine (LGBM) models show the lowest overall accuracy, with the worst performance (33.33%) obtained when using a 60% train–test split ratio and applying different data transformations.

#### 4.8.2. Numerical Experiments for the STFT Approach, with Hyperparameter Optimization

Table 7 presents the top performing AI models with their respective transformations and hyperparameter optimization using the Short-Time Fourier Transform (STFT) approach. The models shown in the table have achieved an accuracy of 80%.

The highest accuracy (88.89%) was achieved by both Gradient Boosting and Random Forest models, where Gradient Boosting used no transformation (NONE) and had 30 training and 45 testing samples, whereas the Random Forest model employed Standard Scaling (STD) with the same number of training and testing samples. Both models had a 60% proportion (proc) of the dataset used.

Additionally, several models, including Support Vector Classifier (SVC), Gradient Boosting, XGBoost (XGB), Gaussian Naive Bayes (GaussianNB), Light Gradient Boosting Machine (LGBM) and Stochastic Gradient Descent (SGD), showed varying levels of performance depending on the transformation and dataset proportions used.

In conclusion, the Gradient Boosting and Random Forest models achieved the highest accuracy in this set of numerical experiments. However, the performance of other models, such as SVC, XGB and LGBM, demonstrated that various transformations and dataset proportions could yield competitive results, warranting further exploration and fine-tuning of these models.

The top performing models with the highest accuracy are the Gradient Boosting and Random Forest models, both without any data transformation (NONE) and 88.89% accuracy. These models were trained on 30 samples and tested on 45 samples, representing a 60% training split.

In the middle range, we find models such as SVC, XGB, and LGBM, with accuracy percentages between 80% and 85%. These models exhibit varying performance depending on the data transformation method applied (STD, MINMAX, or NONE) and the proportion of training data.

Towards the lower end of the performance spectrum, models such as Decision Tree, KNeighbors and SGD show accuracy percentages between 65% and 80%. Similar to the middle-range models, their performance depends on the data transformation method and the proportion of training data.

In summary, the best performing models in this analysis are Gradient Boosting and Random Forest without any data transformation, both achieving 88.89% accuracy. However, the performance of the models is influenced by the data transformation method and the proportion of training data, emphasizing the importance of selecting the appropriate preprocessing techniques and training data splits for a specific task.

#### 4.8.3. Numerical Experiments for the Wavelet Approach without Hyperparameter Optimization

Table 8 presents the results for the third approach. Only the following AI models were able to reach the 80% threshold: Random Forest, Extreme Gradient Boosting, Decision Tree and Gradient Boosting.

The highest accuracy of 94.74% was achieved by the Random Forest model with all three data transformations when the training set size was 37 (50% for training) and the test set size was 38.

The accuracy of the Random Forest model remains consistent at 94.34% for all data transformations when the training set size is 22 (70% for training) and the test set size is 53. Similarly, the model’s accuracy is 93.33% when the training set size is 30 (60% for training) and the test set size is 45.

The Extreme Gradient Boosting model’s highest accuracy of 92.45% was achieved with all data transformations when the training set size was 22 (70% for training) and the test set size was 53.

The Decision Tree models achieved their highest accuracy of 86.67% with all data transformations when the training set size was 30 (60% for training) and the test set size was 45, as well as when the training set size was 45 (40% for training) and the test set size was 30.

The Gradient Boosting model’s highest accuracy of 81.58% was observed with all data transformations when the training set size was 37 (50% for training) and the test set size was 38.

Overall, it appears that the Random Forest model performs the best among the considered AI models, achieving the highest accuracy across different training and testing set sizes and data transformations. It is important to note that the results presented in the table are without hyperparameter optimization, and further fine-tuning might improve the performance of these models.

Since all models achieved different performance for the different configurations, additionally, an average accuracy of the three top performing models was calculated in order to rank them with overall performance in mind, resulting in the following scores:

1. Random Forest: 87.40%.
2. XGBoost: 78.05%.
3. Decision Tree: 76.92%.

Additionally, the following properties can be noted:

1. Transformation methods show no difference in average accuracy across all the models;
2. **Training Set Size Impact:** The average accuracy is generally higher when the training set size is smaller (e.g., 37 or 22), and it decreases as the training set size increases (e.g., 60 or 52);
3. **Testing Set Size Impact:** The average accuracy is generally higher when the testing set size is smaller (e.g., 15, 23, or 30) and lower when the testing set size is larger (e.g., 45, 53).

In conclusion, the Random Forest model performs the best among the tested models, with an average accuracy of 87.40%. There is no significant difference in the performance in regards to the transformation method used. The models tend to perform better when the training set size is smaller and the testing set size is smaller.

#### 4.8.4. Numerical Experiments for the Wavelet Approach with Hyperparameter Optimization

Table 9 shows the results obtained for the final approach. The models that achieved the assumed 80% accuracy threshold include the Gradient Boosting, Random Forest, XGB, LGBM and Decision Tree algorithms.

The highest accuracy of 96.23% was achieved using the Gradient Boosting algorithm with standardization (STD) and 22 training samples. The Random Forest model with STD and 52 training samples closely followed, achieving an accuracy of 95.65%. The Gradient Boosting model with different data transformation methods and training sample sizes consistently achieved high accuracy scores above 90%. The XGB and LGBM models also performed well, especially when used in combination with the MINMAX and STD transformations. The Decision Tree model obtained relatively lower accuracy compared with other models but still managed to achieve results above 80%.

The Gradient Boosting and Random Forest models seem to be the most successful in achieving high scores for this aspect. The results indicate that the choice of data transformation methods and training sample sizes plays a significant role in the performance of these AI models. In many cases, Gradient Boosting and XGBoost models show better performance when combined with either standardization or MINMAX scaling compared with no transformation. However, it is worth noting that there are instances where the models perform similarly with and without transformations. On the other hand, LGBM models tend to have lower accuracies compared with Gradient Boosting and XGBoost models. The Decision Tree model’s performance is more varied, reaching up to 92.45% in some cases, while it stays significantly lower in others.

From these observations, we can conclude that the choice of data transformation methods and the number of training and testing samples significantly impact the performance of AI models. The Gradient Boosting and XGBoost models appear to be more sensitive to these factors, as they generally achieve higher accuracies compared with other models in the table.

## 5. Discussion

### 5.1. Discussion of Numerical Experiments for the STFT Approach without Hyperparameter Optimization

Upon analyzing the table labeled as Table 6, several conclusions can be drawn about the Short-Time Fourier Transform (STFT) approach without any optimization of hyperparameters. It is evident that the Decision Tree model, regardless of the transformation method employed (i.e., MINMAX, STD, or NONE), exhibits exceptional performance, with a 100% accuracy rate when 20% of the data are used for training (n_train=60,n_test=15). This suggests an almost perfect fit of the model to the data in these specific conditions. However, such high performance might also indicate a potential overfitting problem. This aspect needs further evaluation.

Looking further into the results, the Random Forest and XGB models appear to provide a stable accuracy of around 86.84% and 86.79%, respectively, for different proportions of training data and transformations. This performance is consistent, as reflected in instances where 50% of the data (n_train=37,n_test=38) and 70% of the data (n_train=22,n_test=53) are allocated for training. The stability of these models suggests their robustness under various conditions, although they do not achieve the perfect accuracy rate of the Decision Tree model.

In scenarios where the proportion of training data is 60% (n_train=30,n_test=45), both the Random Forest and Decision Tree models present a slightly lower but still substantial accuracy of 86.67%, across all transformation techniques. This demonstrates a minor decrease in performance with an increase in the training dataset size for these models.

The Gradient Boosting model, with an accuracy of 83.02% with 70% of the training data (n_train=22,n_test=53), is slightly less effective than the previously discussed models. The same performance figure is seen for the Random Forest model in these conditions. Moreover, the lowest accuracy observed with the Random Forest model equals 82.61% when the training set is reduced to 30% (n_train=52,n_test=23).

In conclusion, while the Decision Tree model achieved the highest accuracy, its perfect score raises questions about potential overfitting, warranting further investigation. Conversely, the Random Forest and XGB models showed a consistently high level of accuracy across different data proportions and transformations, suggesting reliable performance. Lastly, while Gradient Boosting did not outperform the other models, it still demonstrated a reasonably good accuracy. The impact of hyperparameter optimization on these models could provide more insight and potentially enhance their performance.

### 5.2. Discussion of the Numerical Experiments for the STFT Approach, with Hyperparameter Optimization

The numerical experiments, as presented in Table 7, provide valuable insights into the performance of various models subjected to different data transformations in the context of hyperparameters optimization. The results are both significant and enlightening, and they outline the effectiveness of hyperparameter optimization.

The Gradient Boosting model, when trained with the original dataset (without any transformations), resulted in the highest accuracy, an impressive 88.89%, despite being trained with only 60% of the dataset. A similarly high performance was observed with the Random Forest model subjected to standardization (STD) transformation, reaching the same accuracy level of 88.89% with an identical percentage of training data. These observations highlight the robustness of these models and their capability to efficiently learn from the underlying data, regardless of their size.

The Random Forest model proved to be a consistently high performer, irrespective of the applied transformation or the size of the training set. For instance, it achieved an accuracy of 86.96% for both standard (STD) and no (NONE) transformations, even when trained with just 30% of the data. Such robust performance across varied scenarios signifies the model’s ability to generalize well from the given data.

On the other hand, the Support Vector Classifier model (SVC) demonstrated interesting results. Even though the size of the training set was lowered to 70%, it managed to achieve a fairly consistent accuracy, around 84.91%, irrespective of the applied transformation. These results provide evidence of the SVC model’s resilience against the adverse effects of reduced training data.

The XGBoost (XGB) model, trained with 60% of the data, managed to achieve a similar level of accuracy across different transformations. This again signifies the model’s resilience against different data transformations. However, the Gradient Boosting model appears to have a slight edge over the XGBoost model, as it exhibits higher accuracy under comparable conditions.

The LGBM model also demonstrated consistent performance, achieving 80% accuracy regardless of the transformation and with 60% of the data used for training. On the contrary, the performance of the Stochastic Gradient Descent (SGD) and GaussianNB models, while fairly high, varied depending on the transformation and the size of the training set.

In conclusion, these numerical experiments present a comprehensive view of the relative performances of various models under different transformations and training set sizes. The results clearly demonstrate the benefits of hyperparameter optimization, with the Gradient Boosting and Random Forest models notably standing out. Nevertheless, every model demonstrated a commendable level of accuracy, exceeding the 80% threshold, emphasizing the effectiveness of the Short-Time Fourier Transform (STFT) approach in conjunction with hyperparameter optimization.

### 5.3. Discussion of the Numerical Experiments for the Wavelet Approach without Hyperparameter Optimization

The results of the numerical experiments conducted for the wavelet approach without hyperparameters optimization are presented in Table 8. From these experiments, several notable conclusions can be drawn about the performance of different machine learning models under various conditions, including the effect of data transformation techniques and different proportions of data dedicated to training and testing.

The three models examined include Random Forest, XBoost and Decision Tree, and they were tested with different data transformation methods, namely MINMAX, STD and NONE. In addition, Gradient Boosting was included as a comparison. Furthermore, the proportion of data designated for training was adjusted, with percentages of 50%, 70%, 60% and 40% applied.

Firstly, it can be observed that regardless of the transformation applied or the data partitioning, the Random Forest model consistently performs with high accuracy, exceeding 93%. This suggests that Random Forest is a robust model that maintains strong performance across various transformations and data distributions.

Secondly, the XBoost model also performs admirably, with an accuracy of 92.45% when applied with a training dataset percentage of 70%, regardless of the transformation method used. However, the performance declines to 84.44% when the training data percentage is lowered to 60%. This indicates that the performance of the XBoost model is sensitive to the quantity of training data.

Thirdly, the Decision Tree model shows a decrease in performance compared with the other two models. The accuracy ranges between 84.21% and 86.67% when the training data percentage is at least 50%, while a further decrease is observed as the training data percentage drops to 40% and 70%.

Lastly, the Gradient Boosting model exhibits the lowest accuracy among all models, ranging from 80% to 81.58%. Similar to the Decision Tree model, the Gradient Boosting model’s performance also appears to be sensitive to the proportion of training data.

From this analysis, it can be inferred that both the choice of model and the data distribution (i.e., the percentage split between training and testing data) have a substantial influence on the model’s performance. Furthermore, the data transformation method seems to have a limited effect on the accuracy of these models. Notably, the Random Forest model demonstrates the highest resilience against changes in data distribution and transformation, indicating its potential as a reliable model for this specific task. However, these conclusions warrant further investigation in order to confirm the consistency of the observations under different conditions or datasets.

### 5.4. Discussion of the Numerical Experiments for the Wavelet Approach with Hyperparameter Optimization

In this section, we discuss the insights derived from the numerical experiments performed in the context of the Wavelet approach coupled with hyperparameter optimization. These experiments involved different model types, transformations applied, train–test splits and achieved accuracy rates. The results, as delineated in Table 9, reveal compelling statistical inferences.

First, the Gradient Boosting (GB) model, with a standard deviation (STD) transformation and a training set comprising 70% of the data (n_train=22,n_test=53), achieved the highest accuracy of 96.23%. It can be inferred from this that the GB model, combined with the STD transformation and the mentioned data split, provides a highly accurate prediction.

Next, it can be observed that the Random Forest model, while only employing 30% of the data for training (n_train=52,n_test=23), reached an accuracy close to the best model—95.65%. Despite a smaller training set, the robustness of the Random Forest model combined with the STD transformation resulted in impressive performance.

It was also noticed that the GB model with the STD transformation was consistently effective with different training proportions, delivering an accuracy of 95.56% with 60% of the data for training. When no transformation was applied for the same model, the accuracy marginally decreased to 93.33%, indicating the significance of the STD transformation in optimizing the performance.

An interesting observation can be made regarding the Extreme Gradient Boosting (XGB) model. Regardless of the transformation method applied (or even without using the transformation), and with 70% of the data for training, it constantly yielded an accuracy of 92.45%. This suggests the robustness of the XGB model to variations in data transformations.

In the lower accuracy spectrum, we observe that the Light Gradient Boosting Machine (LGBM) model, irrespective of the transformation method applied or the training split used, attained lower accuracy than the other models. The Random Forest model with minimum–maximum (MINMAX) transformation and 30% training data also fell into the lower accuracy range of 82.61%. Such observations may suggest that these model configurations could be less optimal for the given task.

In conclusion, the insights from this analysis demonstrate the importance of model selection, the impact of data transformations and the balance of the train–test split in model performance. While the GB model with the STD transformation seemed to yield the best results, other models such as Random Forest and XGB also showed robust performances with different configurations. The inferior performance of certain model configurations underlines the need for careful model selection and optimization. As always, these observations should be used as guidance for further experiments and validations.

### 5.5. Summary of Discussion

This paper presented a comprehensive exploration of different machine learning models applied in the context of the Short-Time Fourier Transform (STFT) and Wavelet approaches. Both hyperparameter-optimized and nonoptimized scenarios were explored, offering a broad understanding of the performance dynamics of the employed models.

In the STFT approach without hyperparameter optimization, Decision Tree, Random Forest and XGB models showed remarkable performance. The Decision Tree model achieved the highest accuracy, although its perfect score might indicate a potential overfitting issue, thus necessitating further investigation. The Random Forest and XGB models demonstrated stability and high accuracy levels across different data proportions and transformations. Gradient Boosting, despite being less effective than the former models, still performed reasonably well.

When hyperparameters were optimized in the STFT approach, Gradient Boosting and Random Forest models presented a strong performance. In particular, both models reached an impressive accuracy of 88.89% when trained with 60% of the dataset. The robustness of the Random Forest model was evident, as it performed well across different transformations and sizes of the training set. The SVC and XGB models also showed promising performance, maintaining fairly consistent accuracy levels across different transformations.

In the Wavelet approach without hyperparameter optimization, the Random Forest model demonstrated an exceptional level of accuracy irrespective of the transformation method or data partitioning. The XBoost and Decision Tree models showed a commendable performance, but their accuracy seemed sensitive to the size of the training data. The Gradient Boosting model, on the other hand, displayed the lowest accuracy among all models.

When hyperparameters were optimized in the Wavelet approach, the Gradient Boosting model, combined with the STD transformation and 70% of the data for training, achieved the highest accuracy. The Random Forest model maintained an impressive performance, even when only 30% of the data were used for training. The GB and XGB models showed resilience to transformations, producing high and consistent accuracy figures.

In conclusion, the experimental results underline the importance of an appropriate choice of model, data transformations and data distribution. They suggest that the Random Forest and Gradient Boosting models, under the STFT and Wavelet approaches, respectively, are potentially the most promising candidates for the task at hand, considering their resilience to transformations and robustness across different training sizes. It is important to highlight, however, that these results are based on the current dataset and transformations, and it is always beneficial to carry out further experiments under different conditions to validate these conclusions. Future work could involve a deeper exploration of the optimization techniques and their impact on the models’ performance.

## 6. Conclusions

In this article, a method for tool wear classification is presented and evaluated. The performed tests were based on a set of signals registered during the machining process and measured physical parameters, such as noise or vibrations, saved in separate datasets for each of the used sensors. After the data were collected, the initial signals were processed and prepared for the following operations. Two general methods were used: the first one is based on the Short-Time Fourier Transform, and the second one uses Discrete Wavelet Transform. The hyperparameters were optimized using the exhaustive grid search method. A set of state-of-the-art classifiers was selected in order to further evaluate the consistency of the obtained results in relation to the used parameter values and general experiment setup.

The tested configuration included different types of input data transformations and data splits for training/testing sets for cross-validation. A total of four general approaches were used, each with an associated table showing the best configuration results—the minimal requirement set here assumed that the experiment needed to reach at least an 80% accuracy threshold.

All of the presented configurations show high results, with a significant amount of them exceeding the 90% accuracy threshold and remaining consistent across different configurations. It can also be seen that initial data preparation and used data split or preprocessing methods can influence the results significantly, with some models being more sensitive to such changes than others (as was the case with Gradient Boosting and XGBoost classifiers).

Overall, the presented sensor-based approach achieved more than satisfactory results for some of the parameter configurations, while the general experiments show the impact that various changes in the used parameters or methods for data preprocessing can have on the achieved accuracy. While preparing solutions for any work environments, these factors should be considered. Overall, the impact on the final score can be significant, while each classifier’s susceptibility to such changes is different. Optimizing the used approaches for each problem is a complicated topic, and further research for the best practices in various cases is still required.

Looking towards the future, it is apparent that the quest to identify and establish the best practices in a multitude of scenarios is ongoing. Potential areas for future research include, but are not limited to, the investigation of different sensor-based approaches, enhancement of data preprocessing techniques and further refinement of the classifier parameters. Furthermore, the investigation of other machine learning algorithms that may be more resilient to variations in data preprocessing or changes in parameters could also prove beneficial. It can be anticipated that continual advancements in this field will reveal more effective and efficient solutions for tool wear classification.

## Figures and Tables

**Table 1:** The structure of the data variables in the datasets.

Dataset	Variable	Length of 1 Trial	Sampling Frequency (Hz)	Measure Time (s)
DataHigh	Ac. Emission	27,999,960	5,000,000	5.59
DataLow	Force X	700,000	200,000	3.50
DataLow	Force Y	700,000	200,000	3.50
DataLow	Noise	700,000	200,000	3.50
DataLow	Vibration	700,000	200,000	3.50
DataCurrent	Dev. Current	30,000	50,000	0.60
DataCurrent	Dev. Voltage	30,000	50,000	0.60
DataCurrent	Head Current	30,000	50,000	0.60
DataCurrent	Head Voltage	30,000	50,000	0.60
DataCurrent	Servo Current	30,000	50,000	0.60
DataCurrent	Servo Voltage	30,000	50,000	0.60

**Table 2:** Basic properties of KCR08 cemented carbide.

Carbide Type	Binder Content [%]	Density g/cm^3^	HV10	HV30	HRA	Flexural Strength [MPa]
Submicron chrome	3.2	15.2	1920	1885	93.4	2300

**Table 3:** Basic properties of 50HS spring steel.

Steel Type	Tensile Strength Rm (MPa)	Narrowing (%)	Yield Strength (Re) (MPa)	Hardness (after Softening) (HB)	Hardness (Raw State) (HB)
Chrome–silicon spring	1320	30	1180	269	302

**Table 4:** List of standards used in testing the properties of wood-based materials.

Property	Norm
Static bending strength (MPa)	PN-EN 310
Elasticity modulus (MPa)	PN-EN 310
Screw retention (N/mm)	PN-EN 320
Tensile strength (MPa)	PN-EN 319
Swelling 24 h (%)	PN-EN 317
Water absorption 24 h (%)	PN-D-04234, PN-D-04213, PN-D-04213:1964
Sand content (%)	ISO 3340 (PN-76/D-04245)

**Table 5:** Apparatus used to test the physical and mechanical properties of wood-based materials.

Property	Measuring Apparatus
Density (kg/m^3^)	Laboratory scale, calipers
Static bending strength (MPa)	VebThuringerIndustriewerk SP 10
Elasticity modulus (MPa)	VebThuringerIndustriewerk SP 10
Screw retention (N/mm)	VebThuringerIndustriewerk SP 10
Tensile strength (MPa)	VebThuringerIndustriewerk SP 10
Brinell hardness (HB)	CV Instruments CV-3000LP8
Swelling 24 h (%)	calipers
Water absorption 24 h (%)	calipers
Density profile	GreCon Density Analyzer X-ray

**Table 6:** Top results (with threshold accuracy set at 80%) of numerical experiments for the STFT approach, without hyperparameters optimization.

Model	Transformation	n_train	n_test	% for Training	Acc. [%]
Decision Tree	MINMAX	60	15	20	100.00
Decision Tree	STD	60	15	20	100.00
Decision Tree	NONE	60	15	20	100.00
Random Forest	MINMAX	37	38	50	86.84
Random Forest	STD	37	38	50	86.84
Random Forest	NONE	37	38	50	86.84
XGB	MINMAX	22	53	70	86.79
XGB	STD	22	53	70	86.79
XGB	NONE	22	53	70	86.79
Random Forest	MINMAX	30	45	60	86.67
Random Forest	STD	30	45	60	86.67
Random Forest	NONE	30	45	60	86.67
Decision Tree	MINMAX	30	45	60	86.67
Decision Tree	STD	30	45	60	86.67
Decision Tree	NONE	30	45	60	86.67
Decision Tree	MINMAX	37	38	50	84.21
Decision Tree	STD	37	38	50	84.21
Decision Tree	NONE	37	38	50	84.21
Gradient Boosting	MINMAX	22	53	70	83.02
Gradient Boosting	STD	22	53	70	83.02
Gradient Boosting	NONE	22	53	70	83.02
Random Forest	MINMAX	22	53	70	83.02
Random Forest	STD	22	53	70	83.02
Random Forest	NONE	22	53	70	83.02
Random Forest	MINMAX	52	23	30	82.61
Random Forest	STD	52	23	30	82.61
Random Forest	NONE	52	23	30	82.61
XGB	MINMAX	30	45	60	82.22
XGB	STD	30	45	60	82.22
XGB	NONE	30	45	60	82.22

**Table 7:** Top results (with threshold accuracy set at 80%) of numerical experiments for the STFT approach with hyperparameter optimization.

Model	Transformation	n_train	n_test	% for Training	Acc. [%]
Gradient Boosting	NONE	30	45	60	88.89
Random Forest	STD	30	45	60	88.89
Random Forest	MINMAX	52	23	30	86.96
Random Forest	NONE	52	23	30	86.96
Gradient Boosting	MINMAX	37	38	50	86.84
Gradient Boosting	MINMAX	30	45	60	86.67
SVC	STD	22	53	70	84.91
SVC	MINMAX	22	53	70	84.91
SVC	NONE	22	53	70	84.91
SVC	MINMAX	30	45	60	84.44
SVC	STD	30	45	60	84.44
SVC	NONE	30	45	60	84.44
Gradient Boosting	STD	22	53	70	83.02
XGB	MINMAX	30	45	60	82.22
XGB	STD	30	45	60	82.22
XGB	NONE	30	45	60	82.22
Gradient Boosting	NONE	37	38	50	81.58
Random Forest	MINMAX	37	38	50	81.58
SGD	STD	37	38	50	81.58
GaussianNB	STD	22	53	70	81.13
GaussianNB	MINMAX	22	53	70	81.13
GaussianNB	NONE	22	53	70	81.13
XGB	STD	22	53	70	81.13
XGB	MINMAX	22	53	70	81.13
XGB	NONE	22	53	70	81.13
Decision Tree	MINMAX	45	30	40	80.00
LGBM	MINMAX	30	45	60	80.00
LGBM	STD	30	45	60	80.00
LGBM	NONE	30	45	60	80.00
Random Forest	NONE	30	45	60	80.00
SGD	STD	30	45	60	80.00
SVC	MINMAX	45	30	40	80.00
SVC	STD	45	30	40	80.00
Decision Tree	NONE	45	30	40	80.00
SVC	NONE	45	30	40	80.00
Random Forest	MINMAX	60	15	20	80.00

**Table 8:** Top results (with threshold accuracy set at 80%) of numerical experiments for the Wavelet approach without hyperparameter optimization.

Model	Transformation	n_train	n_test	% for Training	Acc. [%]
Random Forest	MINMAX	37	38	50	94.74
Random Forest	STD	37	38	50	94.74
Random Forest	NONE	37	38	50	94.74
Random Forest	MINMAX	22	53	70	94.34
Random Forest	STD	22	53	70	94.34
Random Forest	NONE	22	53	70	94.34
Random Forest	MINMAX	30	45	60	93.33
Random Forest	STD	30	45	60	93.33
Random Forest	NONE	30	45	60	93.33
XBoost	MINMAX	22	53	70	92.45
XBoost	STD	22	53	70	92.45
XBoost	NONE	22	53	70	92.45
Desition Tree	MINMAX	30	45	60	86.67
Desition Tree	STD	30	45	60	86.67
Desition Tree	NONE	30	45	60	86.67
Desition Tree	STD	45	30	40	86.67
Desition Tree	NONE	45	30	40	86.67
Desition Tree	MINMAX	45	30	40	86.67
Desition Tree	MINMAX	22	53	70	84.91
Desition Tree	STD	22	53	70	84.91
Desition Tree	NONE	22	53	70	84.91
XBoost	MINMAX	30	45	60	84.44
XBoost	STD	30	45	60	84.44
XBoost	NONE	30	45	60	84.44
Desition Tree	MINMAX	37	38	50	84.21
Desition Tree	STD	37	38	50	84.21
Desition Tree	NONE	37	38	50	84.21
Gradient Boosting	MINMAX	37	38	50	81.58
Gradient Boosting	STD	37	38	50	81.58
Gradient Boosting	NONE	37	38	50	81.58
Random Forest	STD	45	30	40	80.00
Random Forest	NONE	45	30	40	80.00
Random Forest	MINMAX	45	30	40	80.00
Gradient Boosting	MINMAX	60	15	20	80.00
Gradient Boosting	STD	60	15	20	80.00
Gradient Boosting	NONE	60	15	20	80.00

**Table 9:** Top results (with threshold accuracy set at 80%) of numerical experiments for the Wavelet approach with hyperparameter optimization.

Model	Transformation	n_train	n_test	% for Training	Acc. [%]
Gradient Boosting	STD	22	53	70	96.23
Random Forest	STD	52	23	30	95.65
Gradient Boosting	STD	30	45	60	95.56
Gradient Boosting	MINMAX	37	38	50	94.74
Gradient Boosting	MINMAX	30	45	60	93.33
Gradient Boosting	NONE	30	45	60	93.33
XGB	MINMAX	22	53	70	92.45
Decision Tree	MINMAX	22	53	70	92.45
XGB	STD	22	53	70	92.45
XGB	NONE	22	53	70	92.45
Gradient Boosting	MINMAX	22	53	70	90.57
Gradient Boosting	NONE	22	53	70	90.57
LGBM	MINMAX	37	38	50	89.47
Gradient Boosting	STD	37	38	50	89.47
Decision Tree	STD	37	38	50	89.47
LGBM	STD	37	38	50	89.47
Gradient Boosting	NONE	37	38	50	89.47
LGBM	NONE	37	38	50	89.47
Decision Tree	NONE	30	45	60	88.89
Random Forest	NONE	52	23	30	86.96
Gradient Boosting	STD	60	15	20	86.67
Decision Tree	NONE	60	15	20	86.67
XGB	MINMAX	30	45	60	84.44
XGB	STD	30	45	60	84.44
Decision Tree	STD	30	45	60	84.44
XGB	NONE	30	45	60	84.44
LGBM	MINMAX	22	53	70	83.02
LGBM	STD	22	53	70	83.02
Decision Tree	NONE	22	53	70	83.02
LGBM	NONE	22	53	70	83.02
Random Forest	MINMAX	52	23	30	82.61
LGBM	MINMAX	30	45	60	82.22
LGBM	STD	30	45	60	82.22
LGBM	NONE	30	45	60	82.22
XGB	MINMAX	37	38	50	81.58
XGB	STD	37	38	50	81.58
XGB	NONE	37	38	50	81.58
Decision Tree	NONE	37	38	50	81.58
Random Forest	NONE	37	38	50	81.58
Random Forest	MINMAX	30	45	60	80.00
Random Forest	STD	30	45	60	80.00
LGBM	MINMAX	45	30	40	80.00
LGBM	STD	45	30	40	80.00
Gradient Boosting	NONE	45	30	40	80.00
LGBM	NONE	45	30	40	80.00
Random Forest	STD	60	15	20	80.00

## Data Availability

Not applicable.
